# Mass Spectrometry Proteomics Characterization of Plasma Biomarkers for Colorectal Cancer Associated With Inflammation

**DOI:** 10.1177/11772719241257739

**Published:** 2024-06-20

**Authors:** Víctor Urbiola-Salvador, Agnieszka Jabłońska, Dominika Miroszewska, Weronika Kamysz, Katarzyna Duzowska, Kinga Drężek-Chyła, Ronny Baber, René Thieme, Ines Gockel, Marek Zdrenka, Ewa Śrutek, Łukasz Szylberg, Michał Jankowski, Dariusz Bała, Wojciech Zegarski, Tomasz Nowikiewicz, Wojciech Makarewicz, Agnieszka Adamczyk, Aleksandra Ambicka, Marcin Przewoźnik, Agnieszka Harazin-Lechowska, Janusz Ryś, Katarzyna Macur, Paulina Czaplewska, Natalia Filipowicz, Arkadiusz Piotrowski, Jan P Dumanski, Zhi Chen

**Affiliations:** 1 Intercollegiate Faculty of Biotechnology of University of Gdańsk and Medical University of Gdańsk, University of Gdańsk, Gdańsk, Pomeranian, Poland; 2 3P-Medicine Laboratory, Medical University of Gdańsk, Gdańsk, Pomeranian, Poland; 3 Institute of Laboratory Medicine, Clinical Chemistry and Molecular Diagnostics, Universitätsklinikum Leipzig, Leipzig University, Leipzig, Saxony, Germany; 4 Leipzig Medical Biobank, Leipzig University, Leipzig, Saxony, Germany; 5 Department of Visceral, Transplant, Thoracic and Vascular Surgery, University Hospital Leipzig, Leipzig, Saxony, Germany; 6 Department of Tumor Pathology and Pathomorphology, Oncology Center‒Prof. Franciszek Łukaszczyk Memorial Hospital, Bydgoszcz, Kuyavian-Pomeranian, Poland; 7 Department of Obstetrics, Gynaecology and Oncology, Collegium Medicum in Bydgoszcz, Nicolaus Copernicus University in Torun, Bydgoszcz, Kuyavian-Pomeranian, Poland; 8 Surgical Oncology, Ludwik Rydygier’s Collegium Medicum in Bydgoszcz, Nicolaus Copernicus University in Torun, Bydgoszcz, Kuyavian-Pomeranian, Poland; 9 Department of Surgical Oncology, Oncology Center‒Prof. Franciszek Łukaszczyk Memorial Hospital, Bydgoszcz, Kuyavian-Pomeranian, Poland; 10 Department of Breast Cancer and Reconstructive Surgery, Oncology Center‒Prof. Franciszek Łukaszczyk Memorial Hospital, Bydgoszcz, Kuyavian-Pomeranian, Poland; 11 Clinic of General and Oncological Surgery, Specialist Hospital of Kościerzyna, Kościerzyna, Pomeranian, Poland; 12 Department of Tumor Pathology, Maria Skłodowska-Curie National Research Institute of Oncology, Kraków, Lesser Poland, Poland; 13 Laboratory of Mass Spectrometry-Core Facility Laboratories, Intercollegiate Faculty of Biotechnology University of Gdańsk and Medical University of Gdańsk, University of Gdańsk, Gdańsk, Pomeranian, Poland; 14 Department of Immunology, Genetics and Pathology and Science for Life Laboratory, Uppsala University, Uppsala, Uppland, Sweden; 15 Department of Biology and Pharmaceutical Botany, Medical University of Gdańsk, Gdańsk, Pomeranian, Poland; 16 Faculty of Biochemistry and Molecular Medicine, University of Oulu, Oulu, North Ostrobothnia, Finland

**Keywords:** Plasma proteomics, colorectal cancer, biomarker, mass spectrometry, complement cascade, diagnosis, inflammation, cancer progression

## Abstract

**Background:**

Colorectal cancer (CRC) prognosis is determined by the disease stage with low survival rates for advanced stages. Current CRC screening programs are mainly using colonoscopy, limited by its invasiveness and high cost. Therefore, non-invasive, cost-effective, and accurate alternatives are urgently needed.

**Objective and design:**

This retrospective multi-center plasma proteomics study was performed to identify potential blood-based biomarkers in 36 CRC patients and 26 healthy volunteers by high-resolution mass spectrometry proteomics followed by the validation in an independent CRC cohort (60 CRC patients and 44 healthy subjects) of identified selected biomarkers.

**Results:**

Among the 322 identified plasma proteins, 37 were changed between CRC patients and healthy volunteers and were associated with the complement cascade, cholesterol metabolism, and SERPIN family members. Increased levels in CRC patients of the complement proteins C1QB, C4B, and C5 as well as pro-inflammatory proteins, lipopolysaccharide-binding protein (LBP) and serum amyloid A4, constitutive (SAA4) were revealed for first time. Importantly, increased level of C5 was verified in an independent validation CRC cohort. Increased C4B and C8A levels were correlated with cancer-associated inflammation and CRC progression, while cancer-associated inflammation was linked to the acute-phase reactant leucine-rich alpha-2-glycoprotein 1 (LRG1) and ceruloplasmin. Moreover, a 4-protein signature including C4B, C8A, apolipoprotein C2 (APO) C2, and immunoglobulin heavy constant gamma 2 was changed between early and late CRC stages.

**Conclusion:**

Our results suggest that C5 could be a potential biomarker for CRC diagnosis. Further validation studies will aid the application of these new potential biomarkers to improve CRC diagnosis and patient care.

## Introduction

Colorectal cancer (CRC) is the third most incident malignancy and the second most deadly cancer worldwide.^1^ Despite the great advances in CRC treatment with recently developed immunotherapies, about 20% to 25% of diagnosed CRC patients present advanced cancer stages and metastasis that is linked to a 5 year survival rate lower than 10% and low therapeutic response.^2,3^ In contrast, diagnosis at early stages leads to reduced tumor-related mortality and a 90% 5 year survival rate after radical surgical resection.^4^ Apart from the disease stage at diagnosis, CRC prognosis depends on multiple factors such as location, genetic factors, molecular expression profiles, tumor immune infiltration, and inflammation.^3^ The low therapeutic response to immunotherapies such as immune checkpoint inhibitors may be caused by the influence of other non-targeted inflammatory and immunosuppressive mechanisms.^5^ Notably, cancer-associated inflammation is considered a well-established hallmark of cancer, especially in CRC.^6^ Inflammatory modulators including chemokines, cytokines, and growth factors influence the interactions between cancer cells and the tumor microenvironment driving tumor progression and the immune response.^7^ Moreover, CRC progression can promote systemic inflammation impacting other organs and facilitating metastasis.^6^

Currently, the gold standard for CRC prevention is colonoscopy complemented with fecal occult blood tests.^8^ However, colonoscopy is expensive and has poor patient compliance, due to its invasiveness and risks, while stool-based tests have low sensitivity and specificity.^4,9^ Therefore, alternative, non-invasive, cost-effective, and easily measurable CRC screening strategies are urgently needed. Mass spectrometry (MS)-based proteomics approaches have been successfully applied to determine blood-based biomarkers of CRC development and progression.^4^ MS-based proteomics characterization of low-abundance proteins in serum/plasma is limited by the high dynamic range of protein concentrations over 9 orders of magnitude with 99% of the total protein content from only 20 abundant proteins.^10^ However, the technological evolution of high-resolution MS instruments such as time-of-flight (TOF) or Orbitrap provides the possibility to discover blood-based biomarkers with high sensitivity and specificity.^11^

Nowadays, the most common blood protein biomarker used in clinical CRC diagnosis is carcinoembryonic antigen (CEA), but its accuracy requires improvement.^12^ Interestingly, untargeted tandem MS coupled with liquid chromatography (LC-MS/MS) proteomics strategies could discover novel potential CRC biomarkers that can be validated by using targeted MS techniques as well as antibody-based assays.^4^ For instance, proteomics analysis discovered that several SERPIN family members are altered in patients with CRC and adenomatous polyps which were validated as potential diagnostic biomarkers by ELISA.^13^ Moreover, plasma proteomics analysis combined with neural network classification identified 5 candidate biomarkers to distinguish between CRC stages.^14^ Another glycoproteomics study detected novel diagnostic biomarkers including elevated levels of complement C9 and fibronectin improved the diagnostic performance of a commercial CEA CRC biomarker.^15^ In addition, targeted proteomics analysis in a non-metastatic CRC cohort determined a 5 protein signature with efficient discrimination of CRC cases from healthy subjects.^16^ However, despite advances in CRC biomarker discovery and validation by proteomics, further studies are needed in larger cohorts to implement reliable biomarkers in clinical practice.

The aim of this study was to discover novel plasma protein signatures involved in CRC development and progression by untargeted LC-MS/MS proteomics analysis. Importantly, we identified significant changes in plasma protein levels associated with cholesterol metabolism, members of the SERPIN family as well as increased levels of complement cascade proteins in CRC patients versus healthy subjects. Furthermore, high complement C5 levels were confirmed in the validation cohort, being a potential diagnostic CRC biomarker. Plasma protein levels of 11 proteins, including complement C8A and serpin family A member 4 (SERPINA4) were linked to cancer-associated inflammation, while 4 proteins, including C8A and C4B, distinguished early from advanced CRC stages.

## Materials and Methods

### Study cohorts and design

This multi-center retrospective study included 36 patients with CRC surgery (age mean: 66.1 ± 11.6 years; 44.4% male) from June 2019 to April 2021 and 26 healthy subjects (age mean: 61.1 ± 10.5 years; 42.3% male) in the discovery cohort. Included patients were with positive colonoscopy and pathologist-confirmed malignant neoplasm. Patients with prior neoadjuvant therapy administration were excluded from the analysis. 69.4% (25 of 36) of diagnosed patients were with advanced CRC stages (III-IV) according to the Union for International Control of Cancer TNM classification and 30.5% (11 of 36) presented cancer-associated inflammation post-operatively assessed by pathologists. Blood samples of healthy subjects and CRC patients were obtained from Biobank HARC, Medical University of Łódź and the 3P–Medicine Laboratory, Medical University of Gdańsk.^17^ The independent validation cohort included 60 CRC patients (age mean: 61.8 ± 11.4 years; 51.7% male) without neoadjuvant therapy and 44 sex-and-age-matched healthy subjects. Serum samples were obtained from the Leipzig Medical Biobank, Germany and the Bank of Biological Material at Masaryk Memorial Cancer Institute, Czech Republic. The collection of whole blood samples was with sterile BD Vacutainer^®^ K2EDTA tubes or Sarstedt S-Monovette^®^ 2.7 mL, K3 EDTA (LMB) before the CRC resection followed by centrifugation, aliquoting, and storage at -80°C until use.

### Sample preparation for mass spectrometry

Proteins were extracted from plasma samples with lysis buffer (1% SDS, 50 mM DTT, 100 mM Tris-HCl pH 8.0) (Merck KGaA, Darmstadt, Germany) containing phosphatase and protease inhibitors (Thermo Fisher Scientific, Waltham, MA, USA) followed by an incubation at 95°C for 10 minutes. Protein concentrations were determined at 280 nm in a μDrop plate with a Multiskan Thermo Nanodrop. Then, 100 μg of proteins were transferred to Microcon 10 kDa filters (Merck KGaA) and were processed based on the Filter Aided Sample Preparation (FASP) protocol.^18^ Briefly, 3 washes with 200 µl of urea buffer (8 M urea,100 mM Tris-HCl pH 8.5) at 10 000 rcf for 20 minutes at room temperature (RT) were applied to the protein mixtures. Free cysteines were alkylated by incubation in the darkness for 20 minutes at RT with 55 mM iodoacetamide (100 µl) in urea buffer (Merck KGaA). Samples were centrifuged at 10 000 rcf for 15 minutes and washed 3 times with urea (100 µl) and 2 times with digestion buffer (50 mM Tris-HCl pH 8.0). Afterward, the filters were transferred into new tubes and proteins were digested by incubation at 37°C with 1 μg of Sequencing Grade Modified Trypsin (Promega, Madison, WI, USA) in 60 µl of digestion buffer overnight. Then, the elution of peptides was performed with the same centrifugation conditions and washed 2 times with 125 and 100 µl digestion buffer. Next, 0.1% trifluoroacetic acid quenched trypsin activity. Peptide concentrations were measured as previously and 20 μg of peptides were desalted with STop And Go Extraction (STAGE) Tips^19^ in Empore C18 extraction disks (3M, Neuss, Germany). Peptides were eluted with 60% acetonitrile and 1% acetic acid. Desalted peptides were dried in a SpeedVac at 45°C and samples were in storage at −20°C until analysis.

### LC-MS/MS analysis

LC-MS/MS analysis of prepared samples was performed with a TripleTOF 5600+ mass spectrometer (SCIEX, Framingham, MA, USA) and with an EkspertMicroLC 200 Plus System (Eksigent, Redwood City, CA, USA). AB SCIEX Analyst TF 1.6 software was used to control the LC-MS/MS system. Samples were run in triplicates with 1.5 µg injected peptides in each technical replicate. Analyses were in a ChromXP C18CL column (3 μm, 120 Å, 150 mm × 0.3 mm) at 5 µl/minute and 35°C, for 60 minutes with an 11% to 35%. acetonitrile gradient in 0.1% formic acid. TripleTOF 5600+ was set in data-dependent acquisition mode and the m/z range of the TOF MS survey scan was at 400 to 1200 Da with an accumulation time of 250 ms. The selection for collision-induced dissociation (CID) fragmentation was set to a maximum of top 20 precursor ions with +2 to +5 charges. The exclusion of precursor ions from reselection was for 5 seconds after 2 occurrences. Product ions spectra were acquired between 100 and 1800 Da with 50 ms accumulation time.

### MS data analysis

Acquired raw SCIEX files were converted to mzML format with MSConvertGUI 3.0 and analyzed using PeaksStudio Xpro 10.6 software (Bioinformatics Solutions, Waterloo, ON, Canada). Peptide sequence search was against the *Homo sapiens* UniProtKB/Swiss-Prot database (release 2022_03) for trypsin digested peptides with maximum 3 missed cleavages per peptide. Carbamidomethylation was as fixed post-translational modification (PTM), whereas N-terminal acetylation and methionine oxidation as variable PTMs. Peptide and protein identification was with a <1% false discovery rate (FDR). Label-free quantification was performed based on the integration of the peptide areas under the curve (AUC).

### Complement C5 validation

Complement C5 serum concentrations were quantified in the validation cohort by an ELISA kit with a coated antibody to human C5 (Abcam ab125963, Cambridge, UK) commercially available, following manufacturer’s instructions.

### Proteomics data and statistical analysis

Statistical analysis was performed with R (version 4.0.3) (R Foundation for Statistical Computing, Vienna, Austria) in RStudio (version 1.3.1093) (RStudio, PBC, Boston, MA, USA). Data preprocessing was performed by summarization of technical replicates with medians and logarithmic transformation of relative abundances. Proteins with missing values in over 50% of patients and 50% of healthy controls were filtered. Random forest imputation was applied to the remaining missing values with the “missForest” R package (version 1.5) followed by quantile normalization. Differences in protein levels between groups were analyzed by the general linear model regression approach with contrast analysis with the “emmeans” R package (version 1.6.2.1). First, for each protein, a general linear model was generated to fit its expression to determine significant changes in CRC patients compared to healthy volunteers including age as a confounding factor. Then, for each protein expression, a general linear model was generated including only CRC patients with the independent variables inflammation and tumor stage while sex was considered a confounding factor. FDR control was applied with the Benjamini & Hochberg correction. Significant changes were considered with FDR-adjusted *P* value < .05. Point-biserial correlation of protein abundance with inflammation status or tumor stage was calculated with the built-in R function cor.test and correlation was significant with a *P* value < .05. Principal Component Analysis (PCA) was performed using prcomp built-in R function and PCA visualization using “factoextra” R package (version 1.0.7). Functional annotation of biological process and cellular component GO terms was performed by a 2-sided hypergeometric test with FDR correction using the Cytoscape cluGO plugin (version 2.5.7). Pathway enrichment analysis of KEGG terms supported by active subnetworks was applied with the R package “pathfindR” (version 1.6.3) using the STRING database and FDR correction. The generation of graphics was with the R package “ggplot2” (version 3.3.5), with the exception of heatmaps generation by the R package “ComplexHeatmap” (version 2.6.2). The construction of the protein network was with Cytoscape (version 3.8.2) using the STRING database and a 0.7 confidence cut-off.

## Results

### Identification and quantification of the plasma proteome of CRC patients using LC-MS/MS

**Figure 1.**
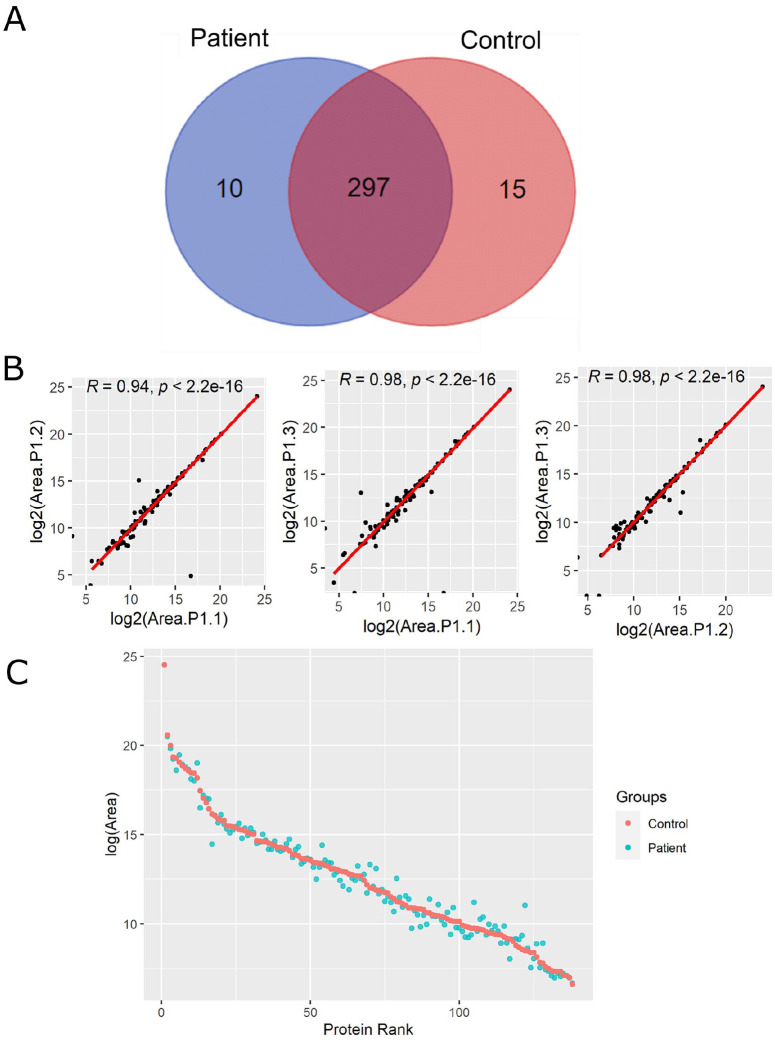
LC-MS/MS analysis of plasma proteome from CRC patients and healthy controls. (A) Venn diagram of identified proteins in CRC patients and healthy individuals. (B) Representative scatter plots of log-transformed areas for the 3 technical replicates from a CRC patient (P1) with their corresponding Pearson correlation coefficients and *P* values. (C) Abundance protein ranking plot with the mean of log-transformed areas from healthy subjects (red) and CRC patients (blue).

To study the protein profile changes in blood involved in CRC development, we applied LC-MS/MS proteomics analysis to plasma samples of 36 CRC patients and 26 healthy controls. As a result, 322 proteins were identified with at least 1 unique peptide with FDR <.01, from which the majority of proteins were identified in both groups (Figure 1A; Supplemental Table S1). Interestingly, IgGFc-binding protein (FCGBP), which is a mucin responsible for innate immune defense in the intestine and is associated with CRC metastasis by promoting cell adhesion, was only identified in CRC patients.^20^

After filtering proteins with high % of missing values, 138 protein groups were quantified. The relative protein abundance was reproducible along technical replicates with high Pearson’s correlation coefficients (Figure 1B). LC-MS/MS analysis quantified proteins in a high dynamic range of concentrations from high-abundance albumin in the range of mg/mL to chemokines such as C-X-C motif chemokine ligand 7 (CXCL7) in the range of ng/mL (Figure 1C).

**Figure 2.**
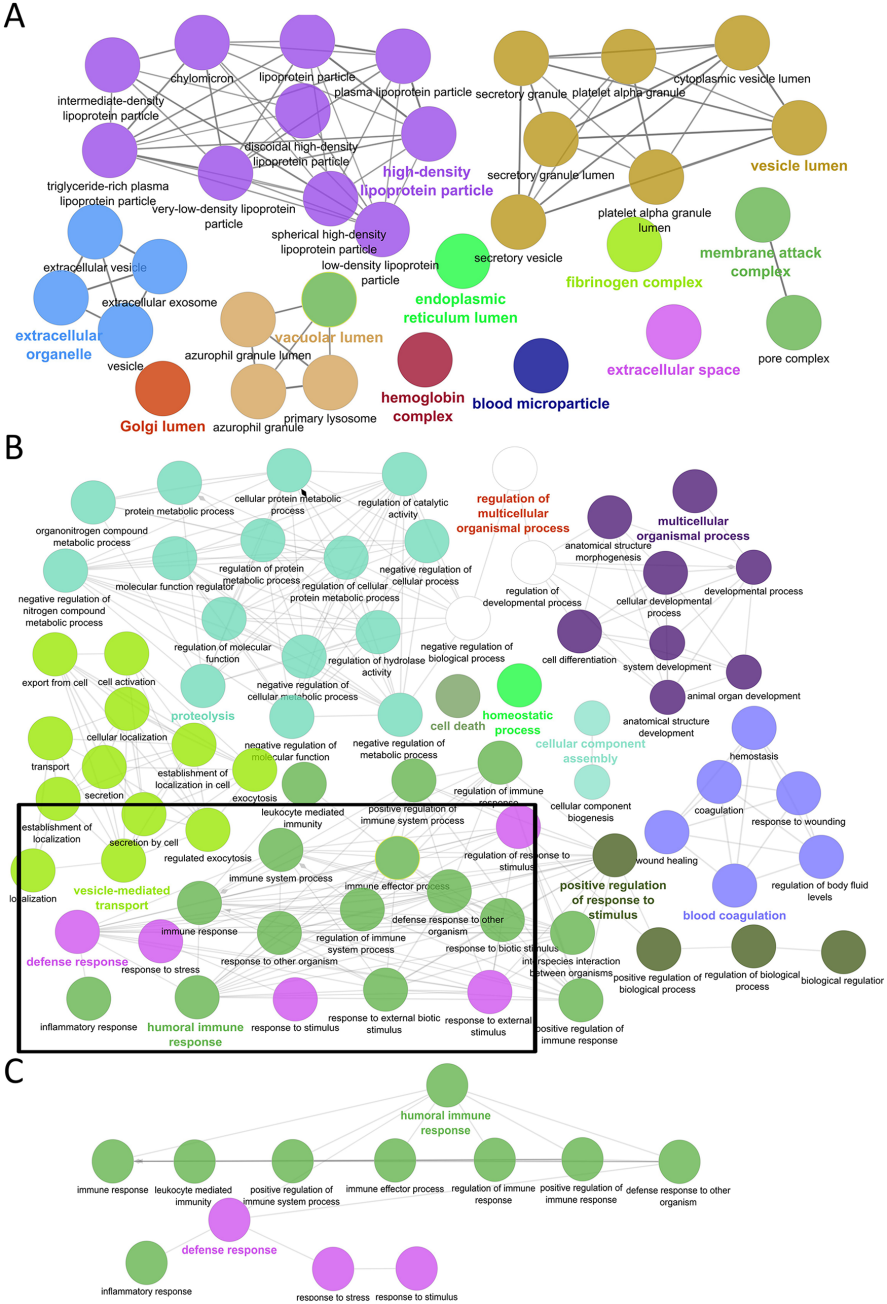
Functional annotation of the identified plasma proteins. (A) Interaction network of over-represented cellular component Gene Ontology (GO) terms with an organic l1ayout. (B) Interaction network of over-represented GO terms of biological processes with an organic layout. (C) Amplification of the subnetwork of GO terms from immune and defense responses with a tree layout.

Functional annotation of the identified proteins determined that the majority were from the extracellular organelles, blood, and lipoprotein microparticles, as well as the vesicle/vacuolar lumen (Figure 2A). However, proteins from the plasma membrane, cytoplasm, and nucleus, such as histone H4, were also detected that may circulate in the peripheral blood due to tissue damage and cell turnover. (Supplemental Table S2). Identified proteins were included in several biological processes such as blood coagulation, homeostasis, proteolysis, and several metabolic processes including cholesterol and fatty acid metabolism, vesicle-mediated transport, cell death as well as humoral immune and inflammatory responses (Figure 2B). Interestingly, over-represented biological process GO terms were associated with different humoral immune and inflammatory responses due to the presence of immunoglobulins, complement proteins, and some chemokines such as CXCL7 (Figure 2C; Supplemental Table S2). Overall, our proteomics analysis identified plasma proteins associated with different biological processes including immune responses and quantified 138 proteins in a high dynamic range of concentrations with high reproducibility.

### CRC development causes protein plasma changes associated with the complement cascade and cholesterol metabolism

**Figure 3.**
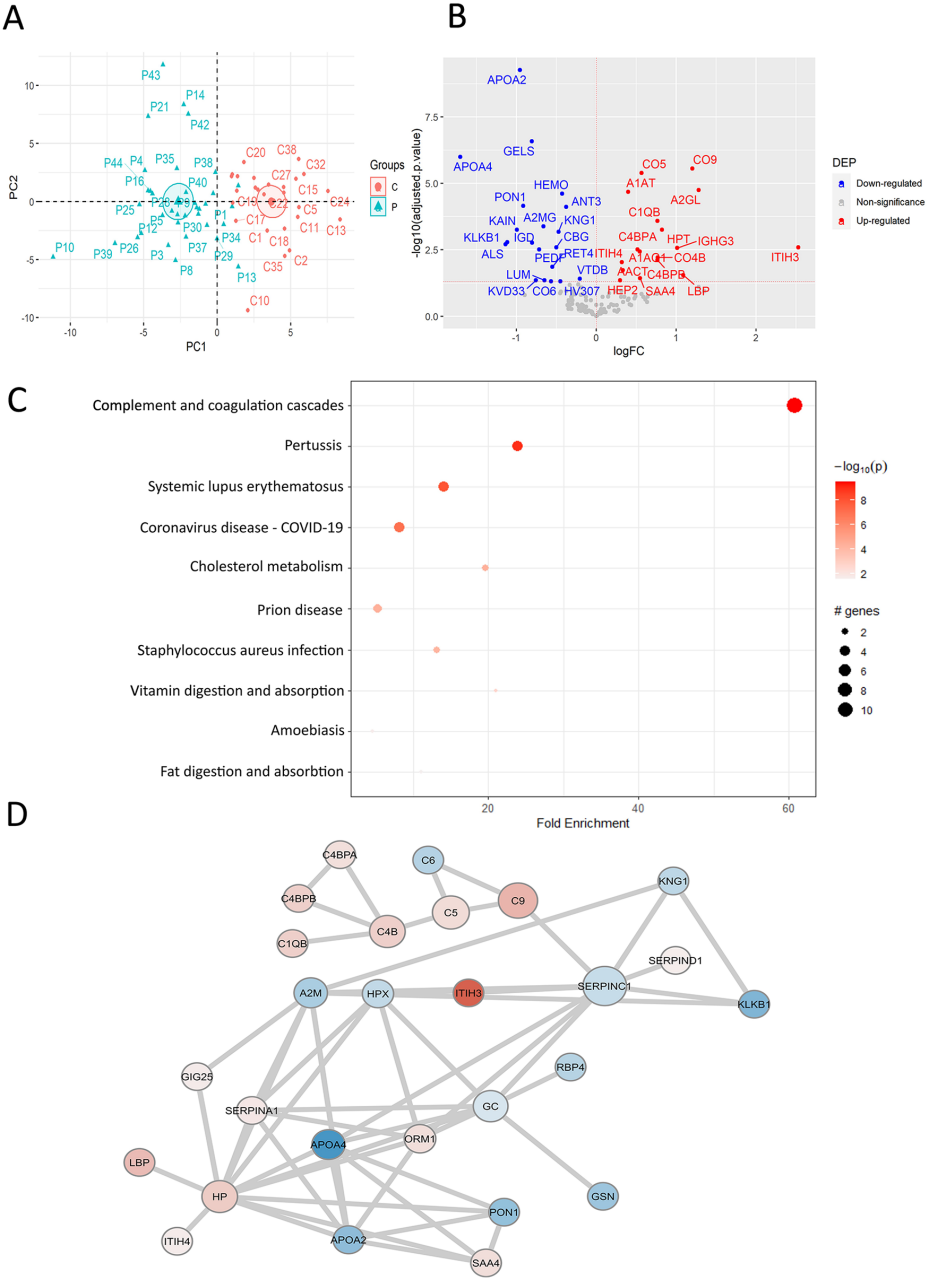
Colorectal cancer (CRC) development causes plasma protein changes involved in complement cascades and cholesterol metabolism. (A) Principal Component Analysis of CRC patients and healthy subjects using the relative abundances of all quantified proteins. (B) Volcano plot of statistical significance against fold-change of proteins between CRC patients and healthy individuals. Colored dots indicate statistically differentially expressed proteins (DEPs) calculated by the general linear model approach. (C) Dot plot of KEGG pathway enrichment combined with STRING protein-protein interaction network analysis from DEPs between CRC patients and healthy subjects. (D) Protein-protein interaction network of DEPs between CRC patients and healthy individuals from STRING database query with a 0.7 confidence cut-off. The size of nodes indicates the degree of connectivity of the nodes. The red and blue dots/nodes represent up-regulation and down-regulation in CRC patients, respectively. FC, Fold Change; p, p-value; PC, Principal Component.

To determine whether the plasma levels of quantified proteins differs in CRC patients versus healthy volunteers, PCA was performed. PCA showed a clear separation of plasma from CRC patients and healthy subjects, indicating that CRC development affects the protein plasma profiles in examined patients (Figure 3A). To unveil these protein changes, differential protein expression analysis was applied, resulting in 17 proteins with enhanced levels and 20 decreased proteins in CRC patients versus healthy volunteers (Figure 3B, Supplemental Table S3). Among the differentially expressed proteins (DEPs), inter-alpha-trypsin inhibitor heavy chain (ITIH)3, leucine-rich alpha-2-glycoprotein (A2GL), C9, and lipopolysaccharide-binding protein (LBP) showed the highest levels in CRC patients, while apolipoprotein (APO) A4, acid labile subunit (ALS), and kallikrein B1 (KLKB1) showed the lowest levels compared to healthy controls. ITIH3, a hyaluronan essential for multiple cellular processes, which transports and regulates hyaluronan turnover in the blood circulation, was found with the highest fold change. Unsupervised hierarchical clustering showed that these 37 DEPs separated CRC from control samples (Supplemental Figure S1).

Pathway enrichment analysis of KEGG terms by active subnetworks revealed that complement and coagulation pathways were activated with elevated protein levels (C4B, C5, C1QB, and C9) in CRC patients (Figure 3C, Supplemental Table S4). Moreover, cholesterol metabolism, vitamin digestion, and adsorption were down-regulated in CRC patients, involving 2 apolipoproteins, APOA2 and APOA4 (Figure 3B and C). Both APOA2 and APOA4 are associated with obesity and hypercholesterolemia that are independent risk factors for CRC development.^21,22^ Similarly, the STRING protein-protein interaction network showed the interaction between the complement proteins with elevated levels (Figure 3D). In addition, SERPINC1 was the most interconnected node linking complement proteins to other DEPs in the network. SERPINC1, also called antithrombin III, is the main inhibitor of blood coagulation which can attenuate inflammatory responses.^23^ Collectively, our analysis indicates that development of CRC causes plasma protein changes which are associated with complement cascade and cholesterol metabolism.

### Plasma protein changes linked to cancer-associated inflammation in CRC patients

**Figure 4.**
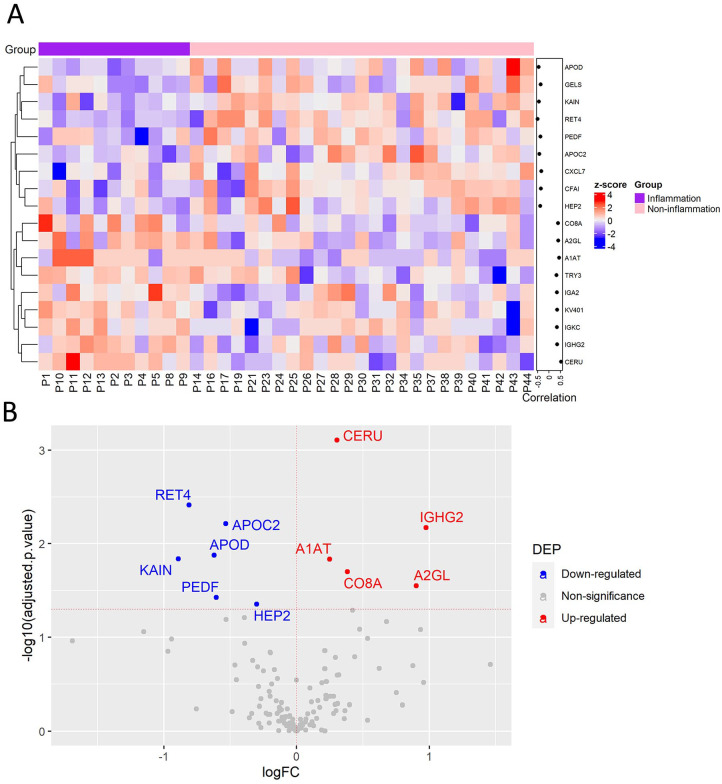
Plasma protein changes induced by cancer-associated inflammation in CRC patients. (A) Heatmap of proteins with significant correlation with inflammatory status. Protein expression is transformed with a z-score by row normalization and distributed by hierarchical clustering. The correlation coefficients (right) indicate a positive/negative correlation for each protein. (B) Volcano plot of statistical significance against fold-change of proteins between CRC patients with inflammation and without inflammation. Dots indicate individual proteins and the red and blue dots represent significant up-regulation and down-regulation in CRC patients with inflammation, respectively.

Inflammation is a well-established hallmark of cancer that influences CRC progression. To analyze protein changes in plasma associated with inflammatory status, the protein levels were compared between CRC patients with cancer-associated inflammation (11 of 36 cases) and without. First, correlation analysis determined significant correlation of 18 proteins with cancer-associated inflammation, including 9 proteins correlated positively such as C8A, A2GL, and ceruloplasmin (CERU), while another 9 proteins including retinol-binding protein 4 (RET4) were correlated negatively (Figure 4A, Supplemental Table S5).

To determine the link between protein abundance and cancer-associated inflammation, the differential protein expression was evaluated by linear regression analysis. This analysis resulted in 11 DEPs that were previously identified with significant correlation (Figure 4B, Supplemental Table S6). Some downregulated proteins were SERPIN family members, for example, SERPINA4 (KAIN) and SERPIND1 (HEP2). Noteworthy, SERPINA4 is an anti-angiogenic and anti-inflammatory agent that was decreased in CRC patients versus healthy volunteers and its downregulation was common in inflammatory processes as well as in cancer.^24^ Additionally, C8A and immunoglobulin heavy constant gamma 2 (IGHG2) may be related to cancer-associated inflammation thus promoting an exacerbated immune response in these patients. Collectively, this analysis determined plasma protein signatures in CRC patients linked to cancer-associated inflammation.

### Evaluation of plasma protein signatures linked to CRC stages

**Figure 5.**
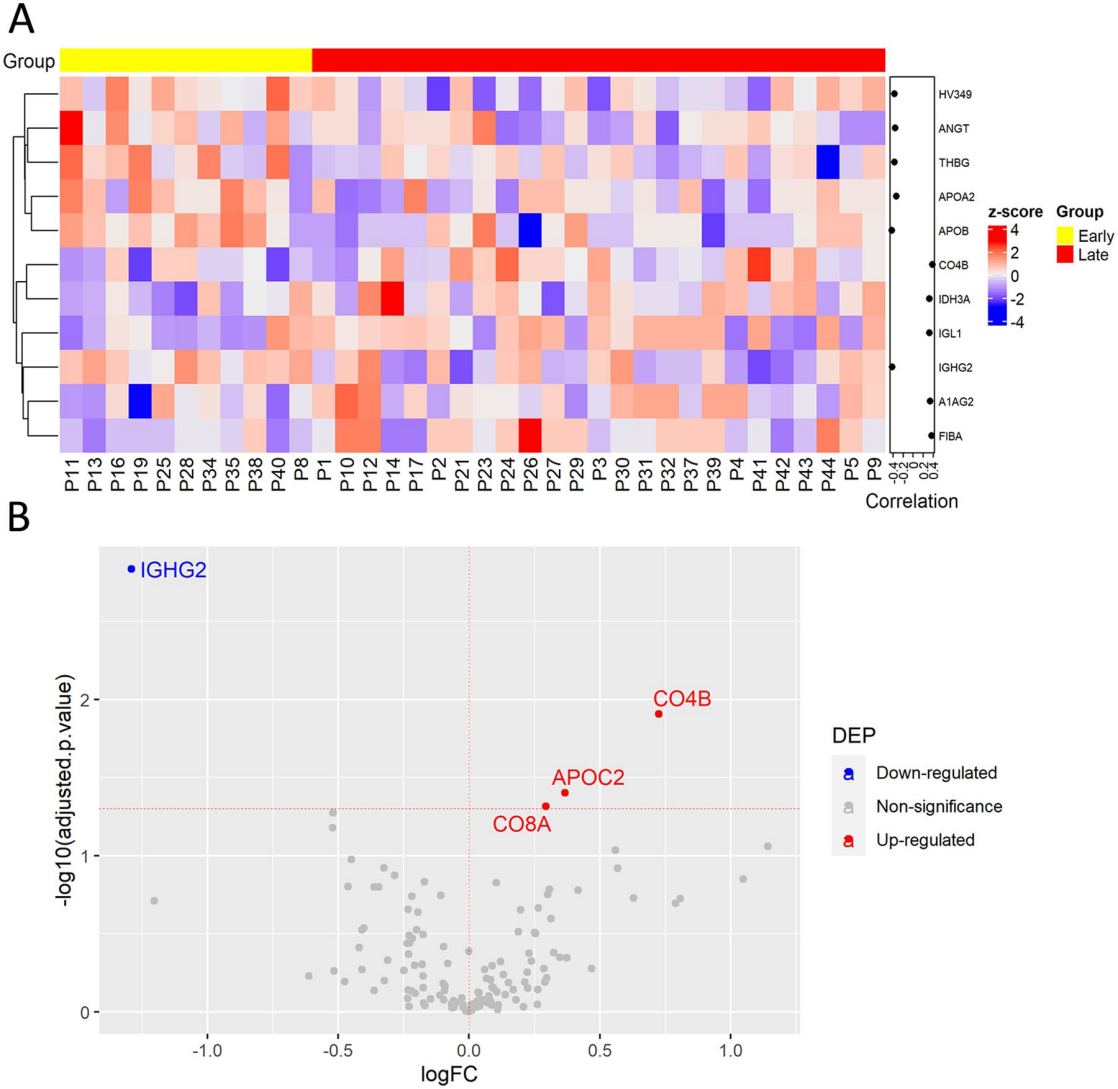
Plasma protein expression differences between early and late stages of CRC. (A) Heatmap of proteins with significant correlation with tumor stage. Protein expression is transformed with a z-score by row normalization and distributed by hierarchical clustering. The correlation coefficients (right) indicate a positive/negative correlation for each protein. (B) Volcano plot of statistical significance against fold-change of proteins between CRC patients with early tumor stage and with late tumor stage. Dots indicate individual proteins and the red and blue dots represent significant up-regulation and down-regulation in CRC patients with late tumor stage, respectively.

The main complication of CRC development is tumor progression and metastasis, resulting in increased CRC mortality. Therefore, CRC prognostic biomarkers are urgently needed. Plasma protein changes linked to CRC progression were determined by comparing protein levels in early-stage patients (I and II) versus late-patients (III and IV). Correlation analysis indicated that 5 proteins were correlated positively, while 6 proteins were correlated negatively (Figure 5A, Supplemental Table S7). Among them, enhanced fibrinogen alpha chain (FIBA) levels in late CRC stages and their association with distant metastasis were previously reported.^25^ Also, increased alpha-1-acid glycoprotein 2 (A1AG2) was linked to shorter survival rates in a CRC cohort.^26^ Similar to the previous comparison, the regression analysis showed that only were 4 DEPs (Figure 5B, Supplemental Table S8). Among them, C8A and C4B may play a relevant role in CRC progression, while the immunoglobulin IGHG2 may be associated with the immune response in CRC early stages by promoting inflammation as enhanced levels were linked to cancer-associated inflammation. Taken together, we found 4 potential biomarkers that can potentially discriminate early from late CRC stages.

### Complement protein C5 plasma levels are enhanced in CRC patients

**Figure 6.**
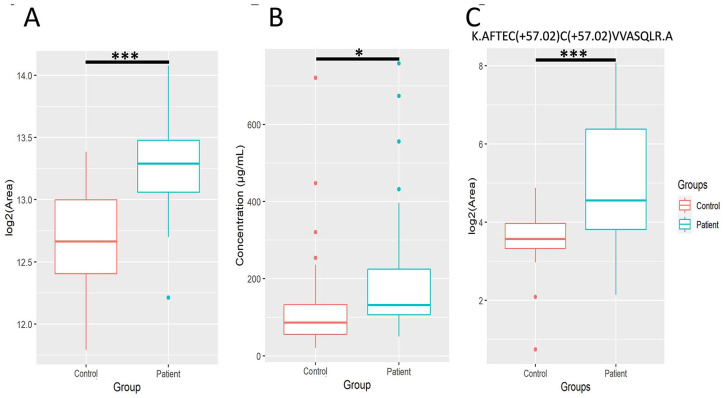
Complement protein C5 is a potential diagnostic biomarker for CRC. Box and whisker plots of (A) log-transformed areas of C5 in the discovery cohort calculated the significance by general linear model approach, (B) C5 concentrations measured by ELISA in the validation cohort calculated by Student t-test, and (C) log-transformed areas of a quantified peptide from C5a with the sequence AFTECCVVASQLR in the discovery cohort for CRC patients and healthy subjects calculated by Student t-test. * indicates statistical significance with a *P* value < .05, and *** indicates a *P* value < .001.

Among the complement proteins, we found elevated C5 levels in plasma of CRC patients versus healthy volunteers by LC-MS/MS analysis (Figures 2B and 6A). To validate this finding, C5 concentrations were measured by ELISA in an independent validation cohort, including 60 CRC patients and 44 healthy subjects (Figure 6B). ELISA results confirmed LC-MS/MS findings. In fact, C5 proteolytic degradation promotes the release of the anaphylatoxin C5a that is an inflammatory mediator.^27^ Noteworthy, a peptide from C5a was also enhanced in CRC patient’s plasma (Figure 6C). Collectively, the enhanced plasma level of complement C5 is a novel promising biomarker for CRC diagnosis and may promote release of the pro-inflammatory C5a.

## Discussion

In this study, we performed LC-MS/MS analysis to characterize the protein changes in plasma involved in CRC development by unbiased proteomics characterization of CRC patients and healthy individuals. Not only secreted proteins were detected but also released intracellular proteins from damaged tissues and cell turnover. Moreover, we quantified 138 proteins with high reproducibility and a high dynamic range of concentrations from ng/mL to mg/mL.

Several plasma proteins were identified with significant changes in CRC patients compared to healthy individuals. These findings were consistent with previously published data performed with LC-MS/MS as well as antibody-based techniques including ELISA and Western blot.^13–16,28–30^ For instance, ITIH3, the DEP with the highest fold change, was reported as increased in CRC patients’ serum and serum of a CRC mice model,^14,16,31^ while another study showed opposite results.^28^ Despite the role of ITIH3 in CRC development has not been determined yet, ITIH4 was found upregulated in CRC tissue versus normal-matched tissue and seems to be involved in the extracellular matrix remodeling and the systemic inflammatory response during CRC development.^28^ Moreover, an increased level of several SERPIN family members was observed in the examined CRC cohort, which is consistent with previously reported data.^13,29^ Among them, SERPINC1 might play a central role in the systemic response to CRC as it is the most interconnected node in the protein-protein interaction network. Moreover, SERPINC1 downregulation may avoid its suppressive tumor activity and inhibit tumor angiogenesis and proliferation.^13^ Interestingly, another family member, SERPINF1 also revealed a link to cancer-associated inflammation. It was reported that this antiangiogenic protein was downregulated in CRC tissue and sera and its low levels were associated with a poor survival prognosis.^30^

Importantly, in this study, the increased level of the complement cascade and its components were found in CRC patients. This indicates that these proteins might play a relevant role in CRC development. Enhanced level of the complement proteins such as C9,^15^ complement component 4 binding protein alpha and beta (C4BPA and C4BPB)^13,32^ was previously reported in CRC patients while increased C1QB is novel. C1QB was found upregulated in tumor tissue versus normal-matched tissue but not in CRC patients’ plasma.^33^ Another novel complement protein with enhanced plasma level is C4B, which is a non-enzymatic component of C3/C5 convertases and was reported as upregulated in the serum of *Apc*^Min/+^ CRC mice versus wild-type mice.^31^ In our study, increased C4B was found in advanced-stage CRC patients, suggesting that this complement protein might play a key role in the disease progression. In addition to C4B, another member of the complement cascade, C8A, was also enhanced in the advanced stages of CRC patients. C8A is a key constituent of the membrane attack complex that regulates the pore formation in target cells and regulates the underlying innate and adaptive immune responses.^27^ The high *C8a* expression was previously reported in CRC metastasis compared to the primary tumor which supports its potential role in CRC progression.^34^ Moreover, the C8A level was also enhanced in patients with cancer-associated inflammation, suggesting that this complement protein is linked to the systemic inflammation promoted by CRC to facilitate metastasis from the primary tumor. More importantly, enhanced C5 was found in CRC patients’ plasma, which was confirmed in the validation cohort. Increased C5 expression in colon tissue versus normal-matched tissue and its association with metastasis was recently reported in another study.^34^ Proteomics analysis also revealed an enhanced level of a peptide corresponding to the C5A anaphylatoxin in examined CRC patients. Although there were no previous reports associating C5A with CRC, another complement anaphylatoxin, C3A, was proposed as a potential CRC diagnostic biomarker.^35^ Moreover, several studies suggest that C5A may promote CRC tumorigenesis, metastasis, and immunosuppressive microenvironment within the tumor.^35–37^ However, further validation studies are needed to confirm the association between C5A plasma levels and CRC. Another enriched pathway in CRC patients was cholesterol metabolism, with 2 downregulated apolipoproteins APOA2 and APOA4, that were previously reported.^38^ It was found that APOA2 polymorphisms were associated with CRC prognosis and might play a relevant role in disease development and progression.^39^ These proteins were also related to metabolic syndrome which is a well-established CRC risk factor.^40^

Interestingly, our analysis reported novel plasma protein changes associated with CRC development. For instance, serum amyloid A4 (SAA4), one of the major acute-phase reactants, was enhanced in CRC patients versus healthy individuals. The increased circulating levels of SAA have been linked to several inflammatory conditions including neoplasia.^41^ *SAA4* was only detected in CRC tissue but not in normal tissue, suggesting a potential role in tumorigenesis.^42^ Another enhanced acute-phase response protein was LBP, which promotes cytokine release in response to bacterial lipopolysaccharide.^43^ Noteworthy, our recently published study demonstrated the increased level of several pro-inflammatory cytokines in the same CRC cohort by proximity extension assay.^44^ It was previously found that LBP polymorphisms were associated with CRC susceptibility^45^ and high serum levels were associated with obesity.^46^

Our analysis identified novel links between plasma protein levels in CRC patients and cancer-associated inflammation. The secreted glycoprotein A2GL, also called LRG1, was upregulated in CRC patients with positive inflammatory status and overall CRC patients versus healthy individuals.^13^ LRG1 was also overexpressed in CRC tissue where it induced cancer proliferation.^47^ Hence, it has been suggested that LRG1 plays an important role in CRC progression and may have an exacerbated pro-inflammatory effect in patients with cancer-associated inflammation due to its link to the acute-phase response.^48^ Another enhanced protein in positive-inflammation CRC patients was CERU while higher levels in CRC patients versus healthy individuals were revealed in another study.^49^ The metalloprotein CERU binds copper in plasma and is associated with inflammatory responses by promoting nitric oxide synthase activity and cytokine secretion.^50^ On the contrary, this study found low levels of the retinol-binding protein 4 (RBP4), which is related to cancer-associated inflammation. Downregulation of RBP4 in CRC patients versus healthy individuals in serum and tumor tissue was previously reported.^51^ Other adipokines with antitumorigenic effects such as adiponectin (APOD) was also reduced in cancer patients and RBP4 may play a role in the reduction of inflammation.^52^ A lower level of APOD, a protein associated with cancer-associated inflammation, was also observed in our cohort. This blood transporter was inversely correlated with CRC tumorigenesis and was associated with early stages of CRC, however, further functional studies are needed to elucidate its role in CRC development.^53^

A comparison early-stage and late-stage CRC patients revealed 4 potential biomarkers associated with cancer progression, including C4B, C8A, APOC2, and IGHG2. The lipoprotein metabolism regulator, APOC2, was found elevated in advanced stages of cancer for the first time, while it was previously described as a potential biomarker of CRC development.^14^ On the contrary, IGHG2 plasma levels were increased in CRC early stages and in patients with cancer-associated inflammation. The *IGHG2* expression was previously detected enhanced in cancer tissues of CRC patients but not in plasma.^54^ Further analysis in larger cohorts will validate our findings to determine the suitability of these potential biomarkers to predict the cancer stage and the association with inflammation.

By using LC-MS/MS proteomics analysis, we quantified 138 plasma proteins in CRC patients and healthy subjects. However, the high dynamic range of proteins limited the quantification of proteins with low abundance. Moreover, due to the relatively low number of patients in the discovery CRC cohort, further validation of the novel potential biomarkers in a larger validation cohort by targeted MS techniques or other quantitative methods such as antibody-based strategies is required. The discovery cohort was also limited by the higher percentage of women, while CRC incidence is higher in men. Finally, CRC family history information and molecular expression profiles of the tumor were missing, which are relevant factors in CRC development and progression.

In this study, LC-MS/MS plasma proteomics application in CRC patients identified novel protein signatures compared to healthy subjects including complement proteins as well as proteins such as SAA4 and LBP associated with pro-inflammatory conditions. Importantly, we confirmed the enhanced levels of C5 in patients of a validation cohort as a potential diagnostic biomarker of CRC. Moreover, several proteins were linked to cancer-associated inflammation and tumor stages that may be prognostic biomarkers after further validation in larger cohorts to apply them in clinics to improve patient care.

## Supplemental Material

sj-pdf-2-bmi-10.1177_11772719241257739 – Supplemental material for Mass Spectrometry Proteomics Characterization of Plasma Biomarkers for Colorectal Cancer Associated With Inflammation Supplemental material, sj-pdf-2-bmi-10.1177_11772719241257739 for Mass Spectrometry Proteomics Characterization of Plasma Biomarkers for Colorectal Cancer Associated With Inflammation by Víctor Urbiola-Salvador, Agnieszka Jabłońska, Dominika Miroszewska, Weronika Kamysz, Katarzyna Duzowska, Kinga Drężek-Chyła, Ronny Baber, René Thieme, Ines Gockel, Marek Zdrenka, Ewa Śrutek, Łukasz Szylberg, Michał Jankowski, Dariusz Bała, Wojciech Zegarski, Tomasz Nowikiewicz, Wojciech Makarewicz, Agnieszka Adamczyk, Aleksandra Ambicka, Marcin Przewoźnik, Agnieszka Harazin-Lechowska, Janusz Ryś, Katarzyna Macur, Paulina Czaplewska, Natalia Filipowicz, Arkadiusz Piotrowski, Jan P Dumanski and Zhi Chen in Biomarker Insights

sj-xlsx-1-bmi-10.1177_11772719241257739 – Supplemental material for Mass Spectrometry Proteomics Characterization of Plasma Biomarkers for Colorectal Cancer Associated With Inflammation Supplemental material, sj-xlsx-1-bmi-10.1177_11772719241257739 for Mass Spectrometry Proteomics Characterization of Plasma Biomarkers for Colorectal Cancer Associated With Inflammation by Víctor Urbiola-Salvador, Agnieszka Jabłońska, Dominika Miroszewska, Weronika Kamysz, Katarzyna Duzowska, Kinga Drężek-Chyła, Ronny Baber, René Thieme, Ines Gockel, Marek Zdrenka, Ewa Śrutek, Łukasz Szylberg, Michał Jankowski, Dariusz Bała, Wojciech Zegarski, Tomasz Nowikiewicz, Wojciech Makarewicz, Agnieszka Adamczyk, Aleksandra Ambicka, Marcin Przewoźnik, Agnieszka Harazin-Lechowska, Janusz Ryś, Katarzyna Macur, Paulina Czaplewska, Natalia Filipowicz, Arkadiusz Piotrowski, Jan P Dumanski and Zhi Chen in Biomarker Insights

## Declarations

**Ethics approval and consent to participate:** This study was performed in line with the principles of the Declaration of Helsinki. Approval was granted by the Independent Bioethics Committee for Scientific Research at the Medical University of Gdansk (NKBBN/443/2021, 2021-06-15 and NKBBN/564/2018, 2018-11-14) and the Bioethics Commission of the Leipzig University (361/21-ek). Informed consent was obtained from all individual participants included in the study.

**Consent for publication:** Not applicable.

**Availability of data and materials:** All data supporting the findings of this study are available within the article and its Supplementary Information. The mass spectrometry proteomics data that supports the findings of this study is available upon request.

**ORCID iDs:** Víctor Urbiola-Salvador  https://orcid.org/0000-0002-8177-2744 Kinga Drężek-Chyła  https://orcid.org/0009-0007-1008-7145

## References

[1] Sung H Ferlay J Siegel RL , et al Global Cancer Statistics 2020: GLOBOCAN estimates of incidence and mortality worldwide for 36 cancers in 185 countries. CA Cancer J Clin. 2021;71:209-249. 33538338 10.3322/caac.21660

[2] Cervantes A Adam R Roselló S , et al Metastatic colorectal cancer: ESMO Clinical Practice Guideline for diagnosis, treatment and follow-up. Ann Oncol. 2023;34:10-32. 36307056 10.1016/j.annonc.2022.10.003

[3] Xi Y Xu P. Global colorectal cancer burden in 2020 and projections to 2040. Transl Oncol. 2021;14:101174. 34243011 10.1016/j.tranon.2021.101174 PMC8273208

[4] Martins BAA de Bulhões GF Cavalcanti IN , et al Biomarkers in colorectal cancer: the role of translational proteomics research. Front Oncol. 2019;9:1284. 31828035 10.3389/fonc.2019.01284 PMC6890575

[5] Jenkins RW Barbie DA Flaherty KT. Mechanisms of resistance to immune checkpoint inhibitors. Br J Cancer. 2018;118:9-16. 29319049 10.1038/bjc.2017.434 PMC5765236

[6] Sirniö P Väyrynen JP Mutt SJ , et al Systemic inflammation is associated with circulating cell death released keratin 18 fragments in colorectal cancer. Oncoimmunology. 2020;9:1783046. 32923147 10.1080/2162402X.2020.1783046 PMC7458668

[7] Terzić J Grivennikov S Karin E Karin M. Inflammation and colon cancer. Gastroenterology. 2010;138:2101-2114.e5. 10.1053/j.gastro.2010.01.058 20420949

[8] Ladabaum U Dominitz JA Kahi C , et al Strategies for colorectal cancer screening. Gastroenterology. 2020;158:418-432. 31394083 10.1053/j.gastro.2019.06.043

[9] Young PE Womeldorph CM. Colonoscopy for colorectal cancer screening. J Cancer. 2013;4:217-226. 23459594 10.7150/jca.5829 PMC3584835

[10] Anderson NL Anderson NG. The human plasma proteome: history, character, and diagnostic prospects. Mol Cell Proteomics. 2002;1:845-867. 12488461 10.1074/mcp.r200007-mcp200

[11] Zhao Y Xue Q Wang M , et al Evolution of mass spectrometry instruments and techniques for blood proteomics. J Proteome Res. 2023;22:1009-1023. 36932955 10.1021/acs.jproteome.3c00102

[12] Kankanala VL Mukkamalla SKR. Carcinoembryonic Antigen. StatPearls Publishing;2022. Accessed May 11, 2023. https://www.ncbi.nlm.nih.gov/books/NBK578172/35201700

[13] Peltier J Roperch JP Audebert S , et al Quantitative proteomic analysis exploring progression of colorectal cancer: Modulation of the serpin family. J Proteomics. 2016;148:139-148. 27492143 10.1016/j.jprot.2016.07.031

[14] Ahn SB Sharma S Mohamedali A , et al Potential early clinical stage colorectal cancer diagnosis using a proteomics blood test panel. Clin Proteomics. 2019;16:1-20. 31467500 10.1186/s12014-019-9255-z PMC6712843

[15] Chantaraamporn J Champattanachai V Khongmanee A , et al Glycoproteomic analysis reveals aberrant expression of complement c9 and fibronectin in the plasma of patients with colorectal cancer. Proteomes. 2020;8:26. 32971853 10.3390/proteomes8030026 PMC7564939

[16] Ivancic MM Megna BW Sverchkov Y , et al Noninvasive detection of colorectal carcinomas using serum protein biomarkers. J Surg Res. 2020;246:160-169. 31586890 10.1016/j.jss.2019.08.004 PMC6957232

[17] Filipowicz N Drężek K Horbacz M , et al Comprehensive cancer-oriented biobanking resource of human samples for studies of post-zygotic genetic variation involved in cancer predisposition. PLoS One. 2022;17:e0266111. 10.1371/journal.pone.0266111 PMC8989288 35390022

[18] Wiśniewski JR . Filter aided sample preparation – a tutorial. Anal Chim Acta. 2022;190:23-30. 10.1016/j.aca.2019.08.032 31655642

[19] Rappsilber J Mann M Ishihama Y . Protocol for micro-purification, enrichment, pre-fractionation and storage of peptides for proteomics using StageTips. Nat Protoc. 2007;2:1896-1906. 17703201 10.1038/nprot.2007.261

[20] Liu Q Niu X Li Y , et al Role of the mucin-like glycoprotein FCGBP in mucosal immunity and cancer. Front Immunol. 2022;13:863317. 35936008 10.3389/fimmu.2022.863317 PMC9354016

[21] Domínguez-Reyes T Astudillo-López CC Salgado-Goytia L , et al Interaction of dietary fat intake with APOA2, APOA5 and LEPR polymorphisms and its relationship with obesity and dyslipidemia in young subjects. Lipids Health Dis. 2015;14:106. 26365669 10.1186/s12944-015-0112-4 PMC4568066

[22] Carmena-Ramon R Ascaso JF Real JT , et al Genetic variation at the ApoA-IV gene locus and response to diet in familial hypercholesterolemia. Arterioscler Thromb Vasc Biol. 1998;18:1266-1274. 9714133 10.1161/01.atv.18.8.1266

[23] Hepner M Karlaftis V. Antithrombin. Methods Mol Biol. 2013;992:355-364. 23546728 10.1007/978-1-62703-339-8_28

[24] Sun HM Mi YS Yu FD , et al SERPINA4 is a novel independent prognostic indicator and a potential therapeutic target for colorectal cancer. Am J Cancer Res. 2016;6:1636. 27648355 PMC5004069

[25] Tang L Liu K Wang J , et al High preoperative plasma fibrinogen levels are associated with distant metastases and impaired prognosis after curative resection in patients with colorectal cancer. J Surg Oncol. 2010;102:428-432. 20672316 10.1002/jso.21668

[26] Gao F Zhang X Wang S Zheng C. Prognostic impact of plasma ORM2 levels in patients with stage II colorectal cancer. Ann Clin Lab Sci. 2014;44:388-393. 25361921

[27] Merle NS Church SE Fremeaux-Bacchi V , et al Complement system part I—molecular mechanisms of activation and regulation. Front Immunol. 2015;6:262. 26082779 10.3389/fimmu.2015.00262 PMC4451739

[28] Jiang X Bai XY Li B , et al Plasma inter-alpha-trypsin inhibitor heavy chains H3 and H4 serve as novel diagnostic biomarkers in human colorectal cancer. Dis Markers. 2019;2019:5069614. 31481982 10.1155/2019/5069614 PMC6701429

[29] Wågsäter D Löfgren S Zar N , et al Pigment epithelium-derived factor expression in colorectal cancer patients. Cancer Invest. 2010;28:872-877. 20504225 10.3109/07357901003735675

[30] Ji D Li M Zhan T , et al Prognostic role of serum AZGP1, PEDF and PRDX2 in colorectal cancer patients. Carcinogenesis. 2013;34:1265-1272. 23393224 10.1093/carcin/bgt056

[31] Ivancic MM Huttlin EL Chen X , et al Candidate serum biomarkers for early intestinal cancer using 15N metabolic labeling and quantitative proteomics in the ApcMin/+ mouse. J Proteome Res. 2013;12:4152-4166. 23924158 10.1021/pr400467c PMC3792563

[32] Battistelli S Stefanoni M Lorenzi B , et al Coagulation factor levels in non-metastatic colorectal cancer patients. Int J Biol Markers. 2018;23:36-41. 18409149

[33] Yang Q Roehrl MH Wang JY. Proteomic profiling of antibody-inducing immunogens in tumor tissue identifies PSMA1, LAP3, ANXA3, and maspin as colon cancer markers. Oncotarget. 2018;9:3996-4019. 29423100 10.18632/oncotarget.23583 PMC5790517

[34] Chang H Jin L Xie P , et al Complement C5 is a novel biomarker for liver metastasis of colorectal cancer. J Gastrointest Oncol. 2022;13:2351-2365. 36388659 10.21037/jgo-22-829 PMC9660033

[35] Ajona D Ortiz-Espinosa S Pio R. Complement anaphylatoxins C3a and C5a: emerging roles in cancer progression and treatment. Semin Cell Dev Biol. 2019;85:153-163. 29155219 10.1016/j.semcdb.2017.11.023

[36] Ding P Xu Y Li L , et al Intracellular complement C5a/C5aR1 stabilizes β-catenin to promote colorectal tumorigenesis. Cell Rep. 2022;39:110851. 35649359 10.1016/j.celrep.2022.110851

[37] Piao C Zhang WM Li TT , et al Complement 5a stimulates macrophage polarization and contributes to tumor metastases of colon cancer. Exp Cell Res. 2018;366:127-138. 29551360 10.1016/j.yexcr.2018.03.009

[38] Voronova V Glybochko P Svistunov A , et al Diagnostic value of combinatorial markers in colorectal carcinoma. Front Oncol. 2020;10:832. 32528895 10.3389/fonc.2020.00832 PMC7258084

[39] Vargas T Moreno-Rubio J Herranz J , et al Genes associated with metabolic syndrome predict disease-free survival in stage II colorectal cancer patients. A novel link between metabolic dysregulation and colorectal cancer. Mol Oncol. 2014;8:1469-1481. 25001263 10.1016/j.molonc.2014.05.015 PMC5528602

[40] Esposito K Chiodini P Capuano A , et al Colorectal cancer association with metabolic syndrome and its components: a systematic review with meta-analysis. Endocrine. 2013;44:634-647. 23546613 10.1007/s12020-013-9939-5

[41] Buck M Gouwy M Wang J , et al Structure and expression of different serum amyloid A (SAA) variants and their concentration-dependent functions during host insults. Curr Med Chem. 2016;23:1725-1755. 27087246 10.2174/0929867323666160418114600 PMC5405626

[42] Michaeli A Finci-Yeheskel Z Dishon S , et al Serum amyloid A enhances plasminogen activation: implication for a role in colon cancer. Biochem Biophys Res Commun. 2008;368:368-373. 18237545 10.1016/j.bbrc.2008.01.079

[43] Heumann D Gallay P Betz-Corradin S , et al Competition between bactericidal/permeability-increasing protein and lipopolysaccharide-binding protein for lipopolysaccharide binding to monocytes. J Infect Dis. 1993;167:1351-1357. 8501324 10.1093/infdis/167.6.1351

[44] Urbiola-Salvador V Jabłońska A Miroszewska D , et al Plasma protein changes reflect colorectal cancer development and associated inflammation. Front Oncol. 2023;13:1158261. 37228491 10.3389/fonc.2023.1158261 PMC10203952

[45] Chen R Luo FK Wang YL , et al LBP and CD14 polymorphisms correlate with increased colorectal carcinoma risk in Han Chinese. World J Gastroenterol. 2011;17:2326-2331. 21633598 10.3748/wjg.v17.i18.2326 PMC3098400

[46] González-Sarrías A Núñez-Sánchez MA Ávila-Gálvez MA , et al Consumption of pomegranate decreases plasma lipopolysaccharide-binding protein levels, a marker of metabolic endotoxemia, in patients with newly diagnosed colorectal cancer: a randomized controlled clinical trial. Food Funct. 2018;9:2617-2622. 29770393 10.1039/c8fo00264a

[47] Zhou Y Zhang X Zhang J , et al LRG1 promotes proliferation and inhibits apoptosis in colorectal cancer cells via RUNX1 activation. PLoS One. 2017;12:e0175122. 10.1371/journal.pone.0175122 PMC5380360 28376129

[48] Shirai R Hirano F Ohkura N , et al Up-regulation of the expression of leucine-rich α2-glycoprotein in hepatocytes by the mediators of acute-phase response. Biochem Biophys Res Commun. 2009;382:776-779. 19324010 10.1016/j.bbrc.2009.03.104 PMC7092932

[49] Nayak SB Bhat VR Upadhyay D , et al Copper and ceruloplasmin status in serum of prostate and colon cancer patients. Indian J Physiol Pharmacol. 2003;47:108-110. 12708132

[50] Linder MC. Ceruloplasmin and other copper binding components of blood plasma and their functions: an update. Metallomics. 2016;8:887-905. 27426697 10.1039/c6mt00103c

[51] Fei W Chen L Chen J , et al RBP4 and THBS2 are serum biomarkers for diagnosis of colorectal cancer. Oncotarget. 2017;8:92254-92264. 29190912 10.18632/oncotarget.21173 PMC5696178

[52] Raucci R Rusolo F Sharma A Colonna G Castello G Costantini S. Functional and structural features of adipokine family. Cytokine. 2013;61:1-14. 23022179 10.1016/j.cyto.2012.08.036

[53] Kopylov AT Stepanov AA Malsagova KA , et al Revelation of proteomic indicators for colorectal cancer in initial stages of development. Molecules. 2020;25:619. 32023884 10.3390/molecules25030619 PMC7036866

[54] Boncheva VB Linnebacher M Kdimati S , et al Identification of the antigens recognised by colorectal cancer patients using sera from patients who exhibit a Crohn’s-like lymphoid reaction. Biomolecules. 2022;12:1058. 36008952 10.3390/biom12081058 PMC9406176

