# Supplementary material for: Mass Spectrometry Proteomics Characterization of Plasma Biomarkers for Colorectal Cancer Associated With Inflammation
Source: Biomark Insights. 2024 Jun 20;19:11772719241257739. doi: 10.1177/11772719241257739 (PMC11191626; doi:10.1177/11772719241257739)
Supplement: sj-pdf-2-bmi-10.1177_11772719241257739 – Supplemental material for Mass Spectrometry Proteomics Characterization of Plasma Biomarkers for Colorectal Cancer Associated With Inflammation [file sj-pdf-2-bmi-10.1177_11772719241257739.pdf]

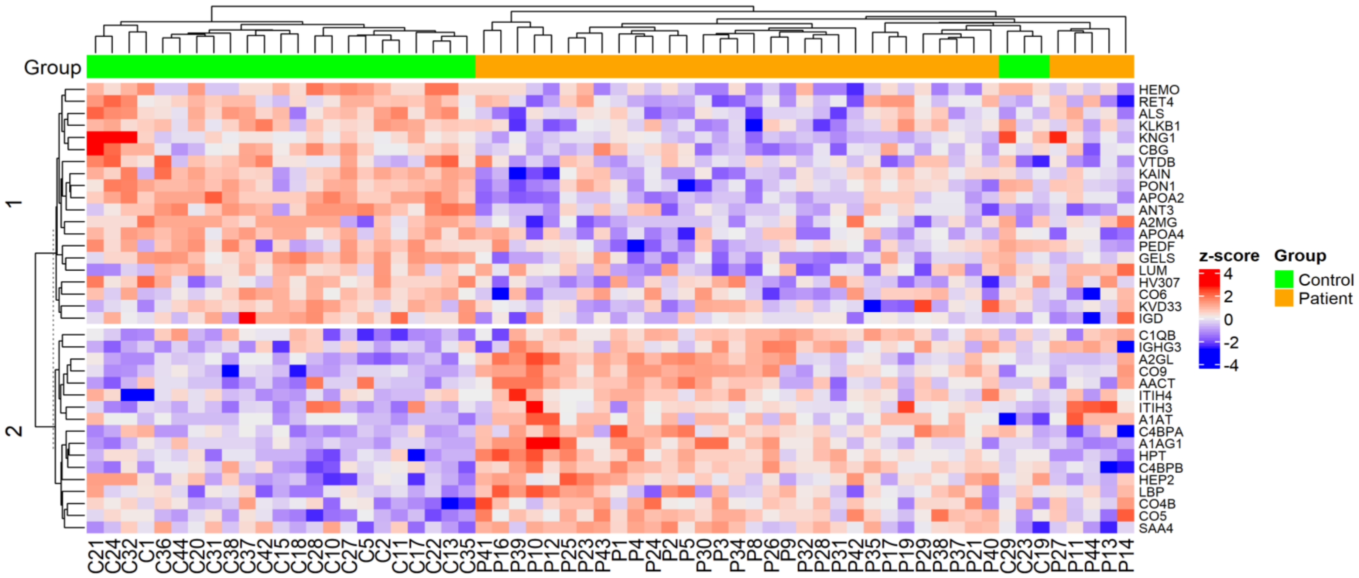

Figure S1. Heatmap of DEPs between CRC patients and healthy subjects with z-score by row normalization and distributed by hierarchical clustering.
